# Evolution of mate-harm, longevity and behaviour in male fruit flies subjected to different levels of interlocus conflict

**DOI:** 10.1186/1471-2148-13-212

**Published:** 2013-09-28

**Authors:** Bodhisatta Nandy, Vanika Gupta, Sharmi Sen, Niveda Udaykumar, Manas Arun Samant, Syed Zeeshan Ali, Nagaraj Guru Prasad

**Affiliations:** 1 Indian Institute of Science Education and Research Mohali, Knowledge City, Sector 81, SAS Nagar, PO Manauli, Mohali 140306, Punjab, India; 2 St. Joseph’s College of Arts and Science, 36 Lalbagh Road, P. B. 27094, Bangalore 560027, Karnataka, India

**Keywords:** Intersexual conflict, Mate-harm, Life-span and aging, Experimental evolution, *Drosophila melanogaster*

## Abstract

**Background:**

Interlocus conflict predicts (a) evolution of traits, beneficial to males but detrimental to females and (b) evolution of aging and life-span under the influence of the cost of bearing these traits. However, there are very few empirical investigations shedding light on these predictions. Those that do address these issues, mostly reported response of male reproductive traits or the lack of it and do not address the life-history consequence of such evolution. Here, we test both the above mentioned predictions using experimental evolution on replicate populations of *Drosophila melanogaster*. We present responses observed after >45 generations of altered levels of interlocus conflict (generated by varying the operational sex ratio).

**Results:**

Males from the male biased (high conflict, M-regime) regime evolved higher spontaneous locomotor activity and courtship frequency. Females exposed to these males were found to have higher mortality rate. Males from the female biased regime (low conflict, F-regime) did not evolve altered courtship frequency and activity. However, progeny production of females continuously exposed to F-males was significantly higher than the progeny production of females exposed to M-males indicating that the F-males are relatively benign towards their mates. We found that males from male biased regime lived shorter compared to males from the female biased regime.

**Conclusion:**

F-males (evolving under lower levels of sexual conflict) evolved decreased mate harming ability indicating the cost of maintenance of the suit of traits that cause mate-harm. The M-males (evolving under higher levels sexual conflict) caused higher female mortality indicating that they had evolved increased mate harming ability, possibly as a by product of increased reproduction related activity. There was a correlated evolution of life-history of the M and F-males. M-regime males lived shorter compared to the males from F-regime, possibly due to the cost of investing more in reproductive traits. In combination, these results suggest that male reproductive traits and life-history traits can evolve in response to the altered levels of interlocus sexual conflict.

## Background

In most sexually reproducing species, males compete for access to females. This competition amongst males can potentially result in male specific adaptations including the ability to manipulate their mates – either physically or physiologically. As a by-product of such manipulation, males often end up causing fitness depression in females [1,2]. Such effect of males on female fitness is generally called mate-harm [3]. Mate-harm in turn selects for increased resistance to male-induced harm in females. The mechanisms of mate-harm vary across species [4,5]. It can range from purely physiological (mediated through chemicals transferred to females during mating) to mechanical (injuries caused during mating). For example, in fruit flies (*Drosophila melanogaster*) mate-harm is caused both by physical coercion during courtship [6-8] and by physiological manipulation mediated through Accessory Gland Proteins [9,10]. Due to these, females suffer mating cost both in terms of fecundity as well as longevity [6-8]. In water striders [11] and bean weevil [12] on the other hand, mate-harm happens principally through mechanical rout, leading to enhanced mortality in females. This dynamic conflict between the two sexes, commonly known as interlocus sexual conflict, can potentially lead to open ended cycles of adaptation and counter adaptation – reminiscent of the “Red queen” dynamics in prey–predator or host-parasite systems [13,14].

Interlocus sexual conflict has been hypothesized to be an important evolutionary force, potentially affecting the evolution of life-history traits [15] and rates of aging [16,17], as well as promoting speciation [18-21].

There are two aspects of interlocus conflict – (a) evolution of male traits related to “mate-harm ability” and (b) evolution of traits related to resistance to male-induced harm in females. Here, we focus on the male part of the conflict. In addition to the evolution of mate-harming ability in males, we also explore the evolution of life-history traits in males from populations routinely experiencing different operational sex-ratios.

A number of experimental evolution approaches have addressed the interesting dynamics related to the evolution of mate-harm and other components of male reproductive behaviour. In one approach, populations were either released from sexual antagonism (by artificially enforcing life-long monogamy) or were subjected to sexual antagonism (by maintaining the normal polygamous mating system). Following several generations of selection, monogamous males were found to be more benign compared to polygamous males [22-25]. Experimentally enforced monogamy has been shown to select for reduced investment in sperm production (i.e., decrease in testes size) relative to the polygamous condition [23,26,27]. These studies indicated the maintenance cost of the relevant male traits. In another approach, populations were subjected to male-limited evolution, wherein males were allowed to evolve with respect to a fixed target female phenotype [28-30]. Both the studies reported increase in male fitness in absence of the gender load. While Rice [28] found harming ability of males to evolve in response to such selection, such response was not observed by Jiang et al. [3]. Yet another approach has been to experimentally evolve populations under different levels of sexual antagonism generated by varying the operational sex ratio of the populations [25,31-34]. In all these studies, females’ ability to resist mate-harm has been found to evolve in response to such selection. Crudgington et al. [25] found males evolved under male biased operational sex ratio to be more harming to their mates relative to males evolved under enforced monogamy. However, Wigby and Chapman [31] found males’ harming ability to be unresponsive to the selection (alteration of operational sex ratio). Thus from the multifarious results observed in a range of experimental evolution studies, it appears that the issue of evolution of mate-harm and male reproductive behaviour and/or physiology under intersexual conflict is far from being settled.

Theories suggest that males under stronger sexual conflict should evolve increased investment in sexual reproduction and related traits at the cost of faster aging and shorter life-span [16,17]. However, only few studies have so far addressed the correlation between sexual conflict and evolution of life-span and aging. Maklakov et al. (2007) and Maklakov and Fricke (2009) did not find any effect of artificially imposed monogamy or polyandry on the life-span and rate of aging of the males of their study populations [35,36]. Thus it is important to test whether evolution under different levels of sexual conflict leads to the predicted [16] changes in life-span and rate of aging.

Here we ask the following questions: (a) Does the ability of males to cause mate-harm evolve under different levels of sexual conflict? (b) If mate-harm evolves under such condition, how do males become more harming? Do their behavioural traits, such as courtship frequency and spontaneous locomotor activity, respond to such selection? (c) Is there a longevity cost to adaptation to varying levels of sexual conflict?

We present the results of an experimental evolution study addressing interlocus conflict. Three replicate populations of *Drosophila melanogaster* were each subjected to three different levels of sexual antagonism by manipulating the operational sex ratio – male biased (M), equal sex ratio (C) and female biased (F). Intensity of interlocus conflict is expected to be high under male biased condition, moderate under equal sex ratio and low under female biased regime. Evolution of sperm competitive ability in these selection regimes has already been reported [37]. We have shown that both components of sperm competitive ability (i.e., offense and defense) have declined under F-regime, while sperm defense has increased under M-regime [37]. Here we report the evolution of (a) body size, (b) harming ability of the males (in terms of both mortality and fecundity), (b) courtship frequency, (c) spontaneous locomotor activity and (d) mean longevity and age specific survival rate under two conditions – reproducing and non-reproducing.

## Results

The experiment was performed using a set of laboratory adapted populations of *Drosophila melanogaster* – LH and LH_st_ see Methods section and [30,38]. The LH_st_ population was split into three replicates (LH_st_ 1–3) and held in the laboratory conditions for three generations. From each of the LH_st_ populations, we derived three sex ratio regimes – Male biased (M1-3, 24 males and 8 females in each vial, 19 vials in each replicate population), Equal sex ratio (C1-3, 16 males and 16 females in each vial, 14 vials in each replicate population) and Female biased (F1-3, 8 males and 24 females in each vial, 19 vials in each replicate population). Thus the experiment consisted of nine populations in all. Due to the method of derivation of these populations (see Methods section), populations with same numerical subscript were treated as statistical blocks during analysis and block was always modeled as random factor. Assays were performed after >45 generations of selection followed by one generation of complete relaxation of selection (see Standardization in Methods section).

### Dry body weight

**Figure 1:**
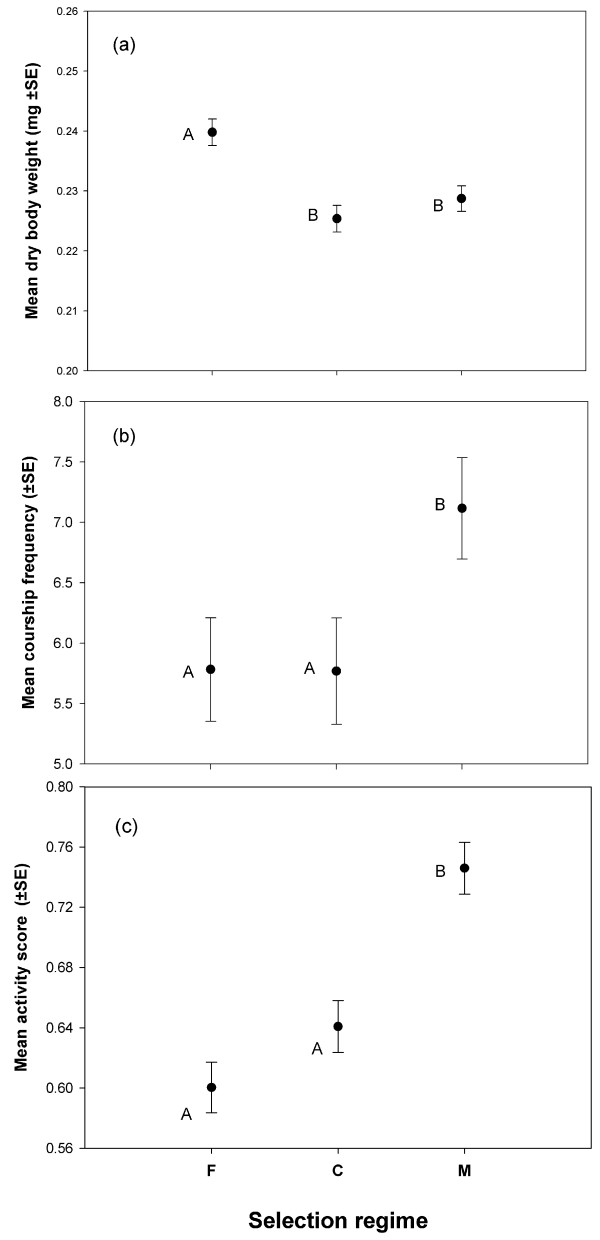
**Response of body size and behavioural traits to selection. (a)** Dry weight at eclosion, **(b)** Courtship frequency and **(c)** Mean activity score of selection regime males. Individuals were weighed in groups of five. An average body weight was calculated using the weight of the five flies. These mean values were taken as the unit of analysis. Mean activity score (see Methods section) and mean courtship frequency were calculated for each vial using the raw data and these were then used as the unit of analysis. Points not sharing common letter are significantly different (determined using Tukey’s HSD).

We measured dry body weight of the freshly eclosed males from the three selection regimes, i.e., M/C/F-regimes (hereafter referred to as “selection regime males” or simply “selected males”). Analysis revealed a significant effect of selection regime (p < 0.0001, Table 1A, Figure 1a). Multiple comparisons using Tukey’s HSD showed that F-males were significantly bigger compared to the males from other two regimes.

**Table 1:** Results of the analyses of body size and behavioural traits

**Trait**	**Effect**	**SS**	**MS Num**	**DF Num**	**MS Den**	**DF Den**	**F**	**p**
(A) Dry body weight	Selection regime	0.003087	0.001544	2	0.000169	4	9.136	**0.032**
	Block	0.001205	0.000603	2	0.000169	4	3.566	0.129
	Selection regime × Block	0.000676	0.000169	4	0.000137	77	1.232	0.304
(B) Locomotor activity	Selection regime	0.292	0.146	2	0.011	4	13.533	**0.016**
	Block	0.034	0.017	2	0.011	4	1.584	0.311
	Selection regime × Block	0.043	0.011	4	0.008	73	1.368	0.253
(C) Courtship frequency	Selection regime	31.149	15.575	2	1.180	4	13.204	**0.017**
	Block	71.673	35.836	2	1.177	4	30.442	**0.004**
	Selection regime × Block	4.700	1.175	4	3.996	78	0.294	0.881

Summary of the results of two-factor ANOVA on (A) dry body weight, (B) locomotor activity and (C) courtship frequency, treating selection regime as the fixed factor crossed with random blocks. Dry body weight was measured in groups of five flies and these values were used as the unit of analysis. For the other two traits, vial means were taken as the unit of analysis. p-values in bold case are statistically significant.

### Mate-harm assay – fitness of the ancestral females exposed to selected males

**Figure 2:**
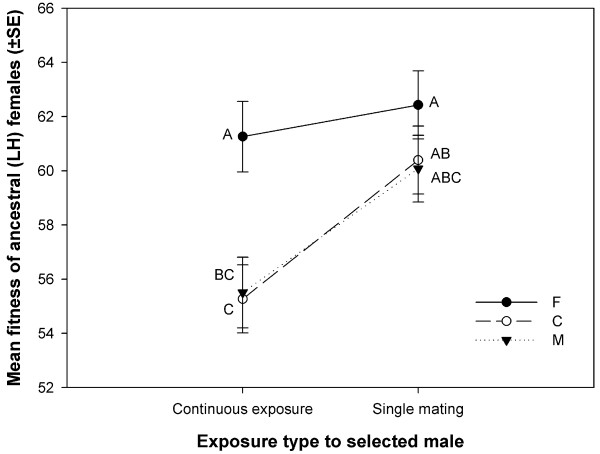
**Harming ability of the selection regime males.** Mean fitness (progeny production) of LH-females under the experimental conditions: after an exposure to the selection line males for one hour (single mating, SM) and two days (continuously exposure, CE). Total number of progeny produced by all the females in a vial was counted and a mean is calculated using this data. The vial means were then used as the unit of analysis. Points not sharing common letters are significantly different (determined using Tukey’s HSD).

To measure the extent of mate-harm caused by the selection regime males, we measured selected males’ effect on ancestral (LH) females’ progeny production during the 18 hour window on 14th day (post egg collection) of their life (a composite measure of female fitness in LH-system). This was done under two conditions – singly mated (SM, females allowed a single mating on 12th day after which they were separated from the males) and continuously exposed (CE, females held with selected males from 12th to 14th day allowing for continuous male harassment of the females). A three factor analysis of variance on the fitness data using selection regime (M/C/F) and treatment (SM/CE) as fixed factors and block (1/2/3) as random factor suggested a significant effect of selection regime (Table 2, Figure 2). Multiple comparison using Tukey’s HSD indicated that females mated to F-males produced significantly more progeny compared to females mated to the males of other two regimes clearly indicating that the F males had evolved to be more benign towards their mates over the two days of interaction. None of the interactions were statistically significant. Compared to females mated once to M or C-males, females continuously held with M or C males produced 8.5% and 8.1% less progeny respectively. When females were held continuously with F males, the decline in progeny production was extremely low (1.8%). However, these differences were not manifest as either a significant effect of mating status or selection regime × mating status interaction.

**Table 2:** Results of the analysis of the data from the mate-harm assay

**Effect**	**SS**	**MS Num**	**DF Num**	**MS Den**	**DF Den**	**F**	**p**
Selection regime	609.96	304.98	2	23.41	4	13.03	**0.018**
Mating status	557.44	557.44	1	90.43	2	6.16	0.131
Block	184.46	92.23	2	67.24	1	1.37	0.525
Selection regime × Mating status	129.32	64.66	2	46.60	4	1.39	0.349
Selection regime × Block	93.60	23.40	4	46.61	4	0.50	0.740
Mating status × Block	180.89	90.45	2	46.61	4	1.94	0.258
Selection regime × Mating status × Block	186.42	46.61	4	45.51	153	1.02	0.397

Summary of results of three-factor ANOVA using selection regime and mating status as fixed factors crossed with random blocks on the progeny production data. Vial means were taken as the unit of analysis. p-values in bold case are statistically significant.

### Longevity assay

We performed a longevity assay on the selected males under two different conditions – non-reproducing and reproducing. In the non-reproducing treatment, selection regime males were collected as virgins and held in single sex-vials for their entire life-span. In the reproducing treatment, selection line males were collected as virgins and two days later were combined with ancestral (LH) females. For both the treatments, male mortality data was collected every alternate day. Mean longevity of the males were calculated using the raw mortality data and subsequently analysed. To measure female survivorship when housed with the selected males, female mortality data was collected in the same way from the reproducing set.

#### Mean longevity

**Figure 3:**
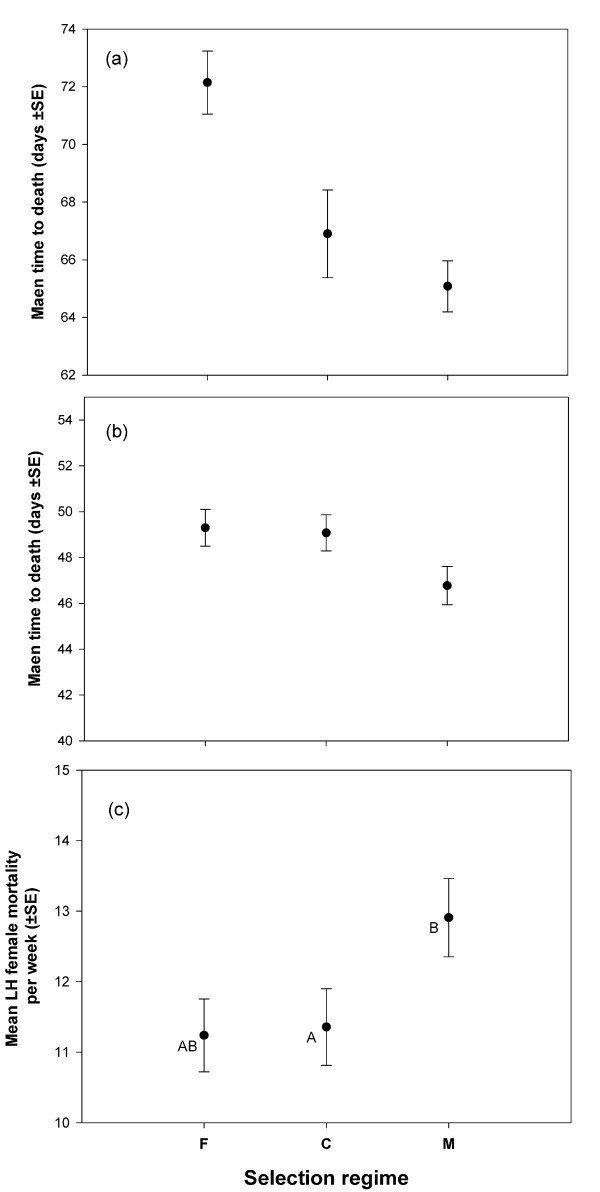
**Results of the longevity assay.** Mean longevity (time to death) of selected males under **(a)** non-reproducing and **(b)** reproducing conditions (continuously held with LH-females). A mean longevity was calculated for each vial. These vial means are used as the unit of analysis. **(c)** LH-female mortality per week. Mortality rate was calculated for each of the nine populations by regressing cumulative weekly mortality across all the replicate vials against time (in weeks). These population level measures of mortality rates were then taken as the unit of analysis. In LH-female mortality rate plot, points not sharing common letter are significantly different (determined using Tukey’s HSD).

The mean longevity of all the three regimes and two treatments were in the range of 46 to 73 days. The maximum longevity ranged from 73 to 99 days. These values are substantially high considering that the flies are maintained on a 14 day discrete generation cycle. Under non-reproducing condition, F-males were found to have higher mean longevity compared to that of M and C-males, while under reproducing condition, M-males had shorter mean longevity compared to both F and C-males (Figure 3a, b). However, the effect of selection regime on the mean longevity under both mating treatments (i.e., reproducing and non-reproducing) was non-significant (Table 3A), very likely due to the heterogeneity of mean longevity of C-males across blocks leading to a nearly significant selection regime × block interaction (Table 3A). Closer scrutiny of the data indicated that C1 behaved differently relative to C2 and C3 under both reproducing and non-reproducing conditions. Hence, we reanalysed the data excluding the C-regime. We observed a significant effect of selection regime on mean longevity under both treatments (Reproducing: p = 0.022; Non-reproducing: p = 0.004, see Table 3B), indicating a significant difference in mean longevity of M and F-males under both treatments. Mean longevity of M-males was significantly lower than the longevity of F-males under both reproducing and non-reproducing conditions (Figure 3a, b).

**Table 3:** Results of the analyses of the data from the longevity assay

**Trait**	**Effect**	**SS**	**MS Num**	**DF Num**	**MS Den**	**DF Den**	**F**	**p**
**(A) Mean longevity including all three regimes**								
Reproducing	Selection regime	117.82	58.91	2	44.26	4	1.33	0.360
	Block	115.23	57.62	2	44.26	4	1.30	0.367
	Selection regime × Block	177.10	44.27	4	18.23	78	2.43	0.055
Non-reproducing	Selection regime	745.96	372.98	2	75.26	4	4.96	0.083
	Block	163.93	81.96	2	75.22	4	1.09	0.419
	Selection regime × Block	301.17	75.29	4	39.28	78	1.92	0.116
**(B) Mean longevity excluding C-regime from analysis**								
Reproducing	Selection regime	95.13	95.13	1	2.28	2	41.69	**0.022**
	Block	263.86	131.93	2	2.27	2	58.14	**0.017**
	Selection regime × Block	4.54	2.27	2	15.78	51	0.14	0.866
Non-reproducing	Selection regime	701.68	701.68	1	2.77	2	253.66	**0.004**
	Block	13.74	6.87	2	2.74	2	2.51	0.285
	Selection regime × Block	5.47	2.74	2	29.35	51	0.09	0.911
**(C) Mortality of LH-females**								
Female mortality	Selection regime	10.69	5.34	2	1.16	4	4.60	0.092
	Block	9.62	4.81	2	1.16	4	4.14	0.106
	Selection regime × Block	4.64	1.16	4	4.70	81	0.25	0.911
Female mortality rate	Selection regime	5.19	2.60	2	0.25	4	10.57	**0.025**
	Block	4.23	2.12	2	0.25	4	8.62	**0.035**

Summary of the result of two-factor ANOVA with selection regime as fixed factor crossed with random blocks on (A) mean longevity under “non-reproducing” and “reproducing” treatments with all three regimes included in the analysis, (B) mean longevity under “non-reproducing” and “reproducing” treatments with C-regime excluded from the analysis, and (C) LH-female mortality - female mortality and mortality rate. Except female mortality rate, vial means were taken as the unit of analysis. Mortality rate of each population was calculated and these population level estimates were used as the unit of analysis. p-values in bold case are statistically significant.

In addition, we performed pair-wise comparisons (i.e., M vs. C; M vs. F; C vs. F) using paired t-tests followed by Dunn-Sidak correction. Mean longevity of each of the population was calculated and these population means were then taken as the unit of analysis. For consistency this was done for both mating status (i.e., reproducing and non-reproducing). The results are summarised in Table 4. Under non-reproducing condition, there was a significant difference between M and F-regimes. Under reproducing condition, the difference between M and F-regimes was marginally not significant (p = 0.02, Dunn-Sidak corrected α=0.017). The differences between C and M or F-regimes were not significant in either mating status (Table 4).

**Table 4:** Results of the pair-wise analyses of mean longevity using paired t-tests

**Mating status**	**Comparison**	**t-Ratio**	**DF**	**Prob > |t|**
**Reproducing**	M vs. F	−6.6451	2	0.02
	M vs. C	1.209229	2	0.35
	F vs. C	−0.06862	2	0.95
**Non-reproducing**	M vs. F	−15.7665	2	**<0.001**
	M vs. C	−2.02121	2	0.18
	F vs. C	0.629118	2	0.59

Population means were taken as the unit of analysis. p-values in bold case are statistically significant.

We also analysed the survivorship data using Cox’s Proportional Hazard model (see Additional file 1). Under non-reproducing condition, males from the M-regime had significantly higher risk ratio compared to males from the F-regime. However we found no significant difference between either M and C or F and C regimes (Table 1A, Additional file 1). Under reproducing condition, males from the M-regime had significantly higher risk ratio compared to males of the C-regime (Table 1A, Additional file 1). However, the difference in survivorship between M and F-regimes was marginally non-significant (Table 1A, Additional file 1) and had significant block effect (Table 2, Additional file 1).

#### Mortality of ancestral females

Total number of female deaths recorded per vial was higher in case of M-males compared to C and F-males, though the difference was non-significant (p = 0.09). Mortality rate of the ancestral females, i.e., number of female deaths observed per week averaged across the male life-span, was found to have significant effect of selection regime of the males (p = 0.02, Table 3C, Figure 3c). Multiple comparisons using paired t-test (paired by blocks) revealed that LH-females died faster when exposed to M-males compared when they were exposed to C-males (t = −7.5, df = 2, p = 0.017, significant after Dunn-Sidak correction). Female mortality rates with F and C-males (t = 0.21, df = 2, p = 0.85), and with F and M-males (t = 4.4, df = 2, p = 0.048) were not significantly different.

### Courtship frequency of selected males

We measured courtship frequency (number of courtship bouts performed by each male per unit time averaged across all observations) of the selection regime males when they were exposed to ancestral (LH) females. This was measured during 3rd and 4th day post eclosion (the natural time of reproduction for these populations). A total of eight observations were taken within this span of time during the light-phase of the Light/Dark cycle. Analysis of the courtship frequency data suggested a significant effect of selection regime (p = 0.024, Table 1C, Figure 1b). Tukey’s HSD indicated that M-males had significantly higher courtship frequency compared to that of C-males (Figure 1b). However, courtship frequency of F-males was not different from that of C-males (Figure 1b).

### Spontaneous locomotor activity

To get an estimate of the overall activity level of the selection regime males, we quantified their spontaneous locomotor activity (proportion of times a randomly chosen individual is seen moving averaged across all observations). This trait has been previously shown to be positively correlated with male fitness [39]. The measurement was done under identical conditions in which courtship frequency was measured. Males were held with ancestral females and observed during the 3rd and 4th day post eclosion (total eight observations). All eight observations were recorded during light phase of the Light/Dark cycle. We found significant effect of our selection on the spontaneous locomotor activity of the selection line males (p < 0.0001, Table 1B, Figure 1c). Through multiple comparisons using Tukey’s HSD, M-males were found to have significantly higher activity score compared to both F and C-males (Figure 1c). Though F-males were less active compared to C-males, the difference was not significant (Figure 1c).

## Discussion

In our study, under decreased intensity of sexual conflict (i.e., F-regime), males evolved to be relatively benign towards females. F-males were found to be have significantly reduced detrimental effect on females’ progeny production, despite evolving larger body size. We did not find them to be less active or less eager in courtship (compared to C-males). The increase in body size of F-males is a likely consequence of relaxed male specific selection in F-regime resulting in the evolution of body size towards the female optima. Such mode of body size evolution has been reported previously in this system [30]. Males evolved under increased level of sexual conflict (i.e., M-regime), tended to be relatively more harming. These males were found to cause significantly higher female mortality over their entire life-span. However, their ability to depress female progeny production was not significantly different from that of the C-males. M-males were found to have increased courtship frequency and spontaneous locomotor activity. In addition, we found the M-males to have shorter mean longevity relative to the F-males under both mating treatments. We now discuss each of these findings in detail.

### Evolution of courtship frequency

We observed an increase in courtship frequency in M-males even when they were held under equal sex ratio. Under male biased operational sex ratio (M-regime), male-male competition is likely to be high and opportunities of mating are likely to be low. As courtship (quality and quantity) is tightly correlated with competitive mating success of males [40-42], selection should favour increased courtship under enhanced male competition for mates. Our results support the observation that courtship frequency increased in *D. pseudoobscura* populations reared at male-biased operational sex ratios for over 50 generations [43]. Holland and Rice [22] found courtship frequency to decrease under experimental removal of sexual selection through enforced monogamy. However, Crudgington et al. [43] did not observe any such decline in courtship frequency in populations of *D. pseudoobscura* evolved under monogamous mating system. As female biased regime is expected to cause a general relaxation of the degree of male-male competition, males from F-regime could in principle evolve decreased courtship frequency. But we did not find any such evidence. One possible reason might be the assay environment, which had equal sex ratio. For F-males, equal sex ratio is a three-fold more male biased condition relative to their normal selection condition. Males in this system are known to show plasticity in the components of their reproductive behaviour in response to varying numbers of competitors [44-46]. Hence, the difference in the selection versus assay condition can potentially explain the observed results.

### Evolution of locomotor activity of males

In a beautifully designed and executed study, Long and Rice [39] showed that ‘adult locomotory activity’ is positively correlated with male fitness. The study also showed that locomotor activity has antagonistic fitness consequences in the two sexes, i.e., it is involved in intra-locus conflict [39]. Under intense competitive condition in M-populations, one would expect a strong selection on male-fitness related traits, such as, locomotor activity. The results confirm this prediction and show a significant increase in spontaneous locomotor activity in M-males compared to C-males. However, we did not observe decline in locomotor activity in F-males relative to C-males, possibly indicating a basal level of selection pressure maintaining male-fitness related traits. Additionally, as discussed in the previous section, *D. melanogaster* males are capable of showing plasticity in their reproductive behaviour based on the number of competitors [44-46]. Since the assay was done under equal sex ratio, which is a relatively more male biased condition than the F-males’ usual maintenance regime, plasticity in male behaviour can contribute to the observed results.

### Evolution of harming ability in males

Mate-harm is a byproduct of a suit of reproductive success enhancing traits in males [4,8,15]. Populations, as simple as laboratory island populations [8], have been found to harbor significant amount of genetic variation with respect to male’s ability to cause such mate-harm [47,48]. A number of previous studies have reported the selection response of mate-harm [22,24,25,28,43]. Enforced monogamy resulted in the evolution of reduced mate harming ability in males (relative to the males under control or polyandrous mating system) either causing less female mortality [24] or being relatively benign to female fecundity [22,25,43]. The ingenious approach of “male-limited evolution” adopted by Rice [28] resulted in the evolution of more competitive males, which also caused more female mortality compared to control males.

However, other studies failed to find any evidence of evolution of mate-harm. Manipulation of intensity of sexual conflict by altering the sex ratio did not cause any evolution in male’s ability to cause harming effects in females [31]. Though Rice (1996) observed evolution of mate-harm using male-limited evolution, a more recent study using the same approach did not see such evolutionary response [3,28].

Our results clearly demonstrate the evolution of mate-harm under altered levels of sexual conflict with M males evolving to be more harming and F males evolving to be less harming to their mates. While males have been shown to harm females in terms of both mortality and life-time fitness [6,7], the natural question our observation raises is – why did our M-males evolve to be harming only in terms of mortality and not in terms of progeny production? The mechanism by which males cause increased mate-harm could be chemical (more toxic ejaculate) or physical (higher amount of courtship). At this point it is difficult to predict whether increase in mate-harming abilities of M-males is due to the evolution of behavioural components or due to the evolution of the ejaculate [37]. Increase in courtship frequency (discussed above) in M-males is an indication of changes in the physical component of mate-harm [6,7]. However, since the change is small, the effect of this was probably only experimentally resolvable under long term exposure rather than short term (2 days) exposure as was done in the mate-harm assay (see Methods, section D).

Under F-condition, males evolved to be relatively benign to females. Females continuously exposed to F-males produced significantly greater number of progeny compared to the females exposed to the males of the other two populations. Even, females which were allowed a single mating with F-males produced more number of progeny compared to those mated to the males of the other two regimes, though this difference was not significant. This indicates that the F-males are benign to their mates, at least in terms of affecting progeny production. Notably, this benign nature of the F-males was evident in spite of them being larger compared to the males of the other regimes (see body size result). At least one previous study has shown that larger males cause more harm (mortality) to females [49]. The fact that the F-males in our study were found to be larger but less harming, clearly underlines the benign nature of these males. Moreover, Rice and Holland [50] have show that evolution of harming ability of males (under enforced monogamy) was not associated with a change in body size. Additionally, since the benign nature of F-males was expressed even after a single round of mating, it is possible that the ejaculate quality and/or quantity of these males have evolved. We did not find any measurable difference in the mortality rate of females held continuously with F-males or C-males. While mate-harm has been shown to affect both female fecundity and longevity [6-8], our finding was not unexpected given that females mated to F-males also produced significantly more number of progeny. Additionally, previous studies about evolution of mate-harm have produced mixed results. While some studies [24,28] have shown evolution in males’ ability to cause mortality in their mates without affecting their fecundity, others have not seen any measurable change in mate harming ability of males [31]. Thus it is possible that evolution of mate harm ability in males in terms of fecundity and survivorship are, at least, to some extent independent of each other.

Our results are different from those of Wigby and Chapman [31] even though the same approach was used to alter the level of sexual conflict. Wigby and Chapman [31] did not find any effect of selection on the harming ability of the males, whereas our results suggest evolution in this trait under both male biased and female biased regimes. This difference in results can possibly be attributed to one major difference in the selection design – collection of virgin flies prior to the setup of adult competition vials and sex ratio treatments. As Wigby and Chapman [31] did not collect virgin flies all the populations experienced similar sex ratio during the first (or more) mating. As a result, in their selection design strength of selection on males is expected to depend on the frequency of mating after the sex ratio regimes are set up. Additionally, some progeny can always be expected to be sired by the males that mated before the sex ratios were set up, diluting the effect of the selection.

Our finding of evolution of mate-harming ability in males is, to some extent, in contrast to that of Jiang et al. [3]. The males from the “male -limited” populations of Jiang et al. (2011) evolved higher fitness but did not show increased mate-harm (in the form of increased mortality of females exposed to males) [3,30]. It is particularly surprising because populations used in our study and that of Jiang et al. [3] share a common ancestry. Jiang et al. [3] cited the possible lack of sufficient additive genetic variation in the ancestral (LH) population surrounding mate-harm related traits as one of the explanations for their result. However, as is evident from our finding, this is not the case. We argue that their finding only suggests that “ML” (male limited) males evolved to reduce the gender load by some mechanism which did not interfere with interlocus conflict. Bedhomme et al. [42], working on the same populations, observed increased efficiency but decreased frequency of courtship activity in males expressing the ML genome compared those expressing C (control) genome. In addition, male limited evolution was associated with decrease in body size (dry weight) [30]. This indicates a decrease in at least the physical component of mate harm. Whereas male-limited evolution “masculinised” the genome without making it more harming, our selection regime directly selected for components of interlocus conflict and led to the evolution of mate-harm. Therefore it is clear that substantial amount of genetic variation still exists in the ancestral population. However in our study, while there was a clear evidence of evolution of mate-harm under F-regime with respect to female fecundity (a measure relevant to the present selection regime), there was a strong trend observed under M-regime with respect to female mortality (which is not a major factor in a population maintained under 14 day discrete generation cycle). Therefore it is possible that the amount of genetic variation is more in one direction (i.e., decrease) than in the other direction (i.e., increase).

Interestingly, along with the evolution of mate-harm, sperm competitive ability has also evolved in our regimes [37]. The F-males, which are relatively benign to their mates have significantly lower sperm defense and offense abilities. On the other hand, M-males, which cause increased mortality in their mates, show significantly higher sperm defense ability. Our findings are consistent with the previous reports which show a positive correlation between males’ sperm competitive ability and mate-harming ability [28,51].

### Evolution of life-span and aging

Sexual conflict has been suggested to have major consequences in the evolution of life-span and aging by affecting baseline investment in reproduction [16,17]. Under high level of male-male competition (increased conflict), males are expected to increase their investment in reproduction, thereby causing the evolution of faster aging and shorter life-span. When sexual conflict is absent or low, populations are thus expected to evolve slower rate of aging and longer life-span. Previous studies have largely ignored the effect of sexually antagonistic adaptations on life-span and aging [17]. Wigby and Chapman [31] looked at the effect of alteration in level of sexual conflict on measures of female longevity but did not address aging in males. In another study, Maklakov et al. [35] observed selection response of seed beetle populations to experimentally enforced monogamy and polygamy and found no effect of selection on male life-span and aging rate.

In the present study, we found M-males to have shorter mean longevity relative to F-males under both reproducing and non-reproducing conditions. There are a number of possible explanations for these observed trends - (a) the trends might represent trade-off between somatic maintenance and reproduction, predicted by the life-history theories of sexual conflict (see above). Increased conflict might have selected for greater investment in reproductive behaviour and/or physiology under M-regime leading to reduced longevity, while the opposite is expected under F-regime. (c) A related possibility is that the males might have evolved different levels of male-male antagonistic interaction (i.e., aggression leading to physical damages and ultimately elevated mortality rates) under the M and F-regimes. This can evolve due to the different levels of male-male competition under the two selection regimes [52]. Under much relaxed intensity of male-male competition, i.e., F-regime, males can potentially evolve to be less aggressive (i.e., reduced male mortality) relative to those evolved under more competitive – M-regime. Such difference between M and F-males can potentially lead to differences in male mortality rates (and mean longevity). However, at this point, in absence of a measure of aggressive behaviour of the selection regime males it is not possible to test this prediction. (b) Alternatively, the increase in body size of the F-males (see Dry body weight discussion) can potentially indicate increased availability of resources for the F-males, leading to the increase in longevity and age specific survival rates. However, as mentioned above, we have found the F-males to evolve reduced harming ability in spite of evolving larger body size, indicating their reduced investment in reproductive behaviour and/or physiology. Therefore at least part of our observation pertaining to the longevity of F-males is very likely to be a reflection of the above mentioned trade-off between reproductive behaviour and/or physiology and longevity. The populations used in our study have been maintained on a 14 day discrete generation cycle for several hundred generations. In such a system, longevity beyond 14th day post egg collection (4–5 days post eclosion) does not contribute to the fitness of the organism and is hence not directly under selection. Hence evolution of longevity beyond 14 days can occur only as a correlated effect of selection. We found that the mean and median longevity in our experiment were in the range of 46 to 73 days, clearly far beyond the usual 14 day maintenance cycle. However, we observed patterns in longevity of the males from the M and F regimes consistent with the predictions of the life-history theories of sexual conflict. Hence, even though the differences in longevity that we observed across the three selection regimes was small, the fact that we observed them at all (with in 50 generations of selection) underscores the importance of sexual conflict in evolution of life span and aging.

## Conclusion

The present study shows the evolution of male traits under altered levels of sexual conflict. We have shown that male courtship frequency and locomotor activity evolve in response to the prevailing level of intersexual conflict and male-male competition. We also provide direct evidence of the evolution of mate-harm in response to the mentioned selection pressure. The trends observed in the longevity and age-specific survival rates were also largely in congruence with the theories of life-history evolution. At least part of our observations can be explained in terms of the body size evolution. Together with our previous report on sperm competitive ability of the selection regime males [37], our study is one of the few experimental evolution studies showing evolution of male traits, under altered levels of sexual conflict through the manipulation of operational sex ratio.

## Methods

### Study population and selection regime

The study was done on nine populations of *Drosophila melanogaster* – M1-3, C1-3 and F1-3 representing male biased, equal and female biased operational sex ratio respectively. These populations were the same as those used in Nandy et al. [37]. All these populations were derived from LH_st_ population [30], which in turn is a derivative of LH population [38]. LH is maintained at a 14 day discrete generation cycle, under 25°C, 60-80% relative humidity, 12 hours light / 12 hours dark (12 hrs: 12 hrs L/D cycle) and on standard cornmeal – molasses – Yeast food, with N_e_ > 5000. They are grown under moderate larval density (140–160 per 8-10 ml of food in 8-dram vials, 25 mm diameter × 90 mm height, 60 vials). On 12th day post egg collection, flies from different vials are mixed and redistributed across fresh food vials seeded with limiting quantity of live Yeast. 16 males and 16 female are kept in these vials. On 14th day, flies are transferred to fresh vials for oviposition and allowed a window of 18 hours. Following this, the adults are discarded and egg densities in these vials are trimmed to 140–160 to start the next generation. LH_st_ was derived by introducing the scarlet eye colour (recessive, autosomal) gene into the LH population [30]. LH_st_ is maintained under a similar condition as LH with N_e_ > 2500. The genetic backgrounds of these two populations are homogenized by periodically back crossing them. LH base population had spent >430 generations (>260 generations for LH_st_) under this laboratory conditions before the start of this selection experiment.

**Figure 4:**
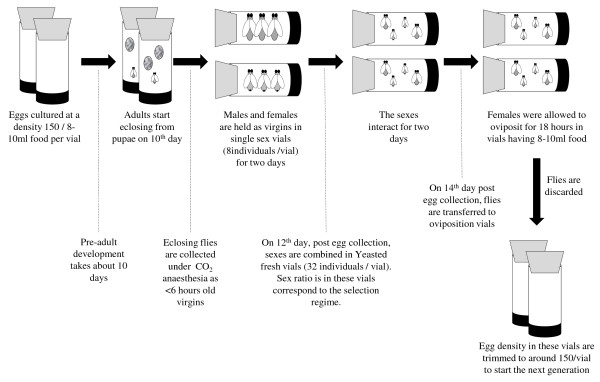
**Maintenance protocol for the selection regimes.** All the populations are maintained under 25°C, ~60% RH, 12 h:12 h Light/Dark cycle on standard cornmeal-molasses-Yeast media in 14-day discrete generation cycles. Larval density is controlled at approximately 150 per vial (8-10 ml of media). On 10th day post egg collection, adults are collected (using CO_2_ anaesthesia) and held as virgins (8 per vial). Two days later adults are combined in ‘yeasted’ vials in the sex ratio corresponding to the respective selection regime. After two days of interaction period, flies are transferred to fresh food vials for oviposition and allowed an oviposition window of ~18 hours. Following the oviposition window, flies are discarded and the egg density in the vials is controlled to start the next generation.

Three replicate populations (C1-3) were derived from LH_st_ and maintained under equal sex ratio for 5 generations. C1-3 differed from the base population with respect to some aspect of the maintenance regime – (a) In C-populations adult flies are collected as virgins on 10th day and held in single sex vials for two days, (b) The sexes are combined on 12th day in fresh food vials seeded with measured amount of live Yeast (7.47 mg per vial, i.e., 0.47 mg per female). We then derived two other populations (M and F) from each of C populations. Thus, populations bearing the same numerical subscript are more closely related to each other by ancestry and are hence treated as statistical blocks. All these populations are maintained as 2-week discrete generation cycle, under 12 hrs:12 hrs L/D cycle at 25°C (±1) temperature and 60-80% relative humidity. Standard corn meal-molasses-Yeast media is used to maintain them. The maintenance protocol is given in Figure 4. Approximately 140–160 eggs are cultured in 8-10 ml of corn meal media per 8-dram vial (25 mm diameter × 90 mm height), referred to as juvenile competition vials. For each population, virgin males and females are collected under light CO_2_ anaesthesia on 10th day (post eclosion). All flies are collected during the peak of eclosion and held as single sex vials at the density of 8 per food-vial. Two days later, on 12th day (post egg collection), sexes are combined following the selection regime – M (24males: 8females in each vial), C (16male:16female in each vial) and F (8male: 24female in each vial). Combination was done in fresh food vials provisioned with measured amount of live Yeast. Amount of Yeast available per female was kept constant across the three selection regime at 0.47 mg / female. These vials (adult competition vials) are kept undisturbed for two days and on 14th day of the cycle, the flies are transferred to fresh food vials (oviposition vials) and allowed to oviposit for 18 hours. After this 18 hour window, flies are discarded and the egg density is trimmed to 140–160 per vial, starting the next generation. Thus the oviposition vials of one generation become the juvenile competition vials of the next generation. The effective population sizes of all the populations are maintained at about 450 following the definition provided by Crow and Kimura [53], N_e_ = 4N_m_N_f_/(N_m_ + N_f_). This method of calculating N_e_ has been adopted by Reuter et al. [54]. An alternative method of calculating N_e_ considering sperm precedence and female multiple mating has been suggested by Rice and Holland [50]. Using this method, N_e_ of all the populations in our experiment was greater than 350. Previous studies [50,54,55] also suggest that in experimental evolution studies such as ours, the effect of differential genetic drift within 45–55 generations of selection (as in our study) is negligible as long as the N_e_ is not critically low. Previous studies by Rice and Holland (2005), Reuter et al. (2008) and Snook et al. (2009) have estimated N_e_ of about 200, 100 and 120–160 respectively [50,54,55]. In all these studies, the effect of differential genetic drift was negligible. Given that N_e_ of all the populations in our experiment was much beyond this size (all N_e_ > 350), differential genetic drift is very unlikely to have played a significant role in our experimental findings.

### Standardization and generation of experimental flies

All populations were passed through one generation of standardization before experimental flies were raised from them. This was done to equalize the potential non-genetic parental effects across the three different regimes [56]. Standardization included maintaining the population under the ancestral culture condition, which does not include virgin collection and sex ratio alteration (see ‘Study population and selection regime” section, LH maintenance protocol). Briefly, in this standardization generation, adults were allowed to grow till 12th day in the juvenile competition vials. On 12th day adults of a given population are mixed across different vials and redistributed across adult competition vials with limiting amount of live Yeast. Two days later, they are transferred to oviposition vials where they oviposited for 18 hours.

After standardization, eggs were collected for the generation of the experimental flies at a density of 130–150 per vial (8-10 ml of cornmeal food). On 10th day after egg collection, males were collected as virgins during the peak of their eclosion and held as single sex vials at an adult density of 10 per vial (for mate-harm assay), 8 per vial (for longevity-assay) and 5 per vial (for courtship frequency assay and locomotor activity assay).

LH females used in this experiment were raised in similar conditions and collected as virgins during the peak of eclosion. Virgin LH-females were held as single sex vials in groups of 8 per vial (for mate-harm assay and longevity assay) and 5 per vial (for courtship and locomotor activity assay). Eggs for LH flies were collected on the same day as that of the selection lines. Thus for all populations the age of the experimental flies were same during the experiment.

### Measurement of dry body weight of selection regime males

This was done after 45–47 generations of selection. Here and in the other assays as well, when assays were spread over multiple generations, all the populations belonging to a given block (e.g., M1, C1 and F1) were assayed together in the same generation. Different blocks were assayed in separate generations. For example, body weight assay was done after 45 generations for Block-1, after 46 generations for Block-2 and after 47 generations of selection for Block-3. Freshly eclosed males were flash-frozen. The frozen flies were dried at 60°C for 48 hours and weighed in a high precision electronic balance (Sartorius CPA225D) to the nearest 0.01 mg. A total of 45–50 males per population were measured for body weight distributed in ten groups of 5 each. Mean body weight of each group was calculated and taken as the unit of analysis.

### Mate-harm assay – progeny production by females exposed to selected males

This assay was also done after 45–47 generations of selection. Fitness (progeny produced) of LH females exposed to the selection line males were assayed under two conditions – singly mated (SM) and continuously exposed (CE). In case of SM, 8 virgin females (2-day old) were transferred into fresh mating vial (seeded with 3.736 mg live Yeast) along with 10 virgin males (2 day old) from one of the nine populations (M1-3, C1-3, F1-3). Combinations were done without anaesthesia. For each population, 18–20 such vials were set up. Of these, 8–10 were randomly assigned to SM and 8–10 to CE. In the SM set, males and females were allowed to interact for one hour. In our flies, this period is just enough to complete a single mating. After one hour, males and females from the SM set were separated under light CO_2_ anaesthesia, males were discarded and the females returned to the same vials. They were then held for two days before oviposition. In the CE set, males and females were allowed to interact continuously for two days. After the two day period, females from the SM and CE sets were transferred to oviposition bottles (Laxbro, FLBT 20, 60 mm diameter × 140 mm height) with ample amount of food (8 females per bottle). Females were allowed a window of 18 hours for oviposition, after which they were discarded. The eggs were incubated at 25°C for 12 days and frozen at −20°C upon complete eclosion of the progeny. The bottles were checked for any sign of crowding. The progeny count was taken as measure of female fitness which can be compared across the different selection regimes. The total number of progeny in each bottle counted and was then treated as the unit of analysis.

### Longevity assay

The longevity assay was done after 50 generations of selection. Longevity of selected males was measured under two conditions – (a) reproducing and (b) non-reproducing. For the “reproducing” set, 8 virgin females (2 day old) from the LH population were combined with 8 males (2 day old) from one of the nine populations (M1-3, C1-3, F1-3) in a vial seeded with 3.74 mg live Yeast. 10 such vials were set up for each of the nine populations. For the “non-reproducing” set, 8 virgin males (2 day old) were transferred to Yeasted vials (3.74 mg) without females. For this set as well, 8–10 vials were set up per population. Flies were transferred to fresh food every alternate day without anaesthesia. Dead flies were sexed and counted during every transfer. For reproducing set, sex ratio was maintained at 1:1 by introducing LH-female(s) (in case of a LH female death) into a vial. Throughout the assay, we never had to remove LH females from any of the vials to equalize sex ratio. This is because female mortality was always higher than male mortality. Hence, none of the LH females left the vials alive. Extra LH females were maintained as a separate set under similar conditions (uncrowded, equal sex ratio, ample food) with LH males. On day 49, we ran out of replacement females (either they were dead or in very bad condition) and thus the replacement had to be stopped.

#### Mean longevity of the selection regime males

We calculated mean longevity of the selection regime males for each vial using the mortality data. During the analysis of mean longevity, these vial means were taken as the unit of analysis. During the entire assay period we also recorded number of death of LH-female in each vial in addition to keeping track of the mortality of selection line males. We used data of the death of ancestral females as an indicator of the mate-harm of the males that they were housed with (see later). We also analysed the mortality data using Cox’s Proportional Hazard model. The details of the method are provided in Additional file 1.

#### LH-female mortality

The design of the longevity experiment allowed us to measure and compare selected males influence on females’ mortality. Throughout the longevity assay, we recorded the deaths of LH-females in the ‘reproducing’-longevity vials. Whenever a LH female died in any of the vials, she was replaced with another LH female held under similar conditions (see above for detail). We explicitly did not use virgin females as replacements as these can severely alter male mating behavior. Mortality of replacement LH-females’ can also be attributed to the effect of LH-males they were held with prior to the actual replacement. However, this is unlikely to affect our observations since individuals from the same group of LH females were used as replacements for all the three selection regimes. Hence, any difference in the mortality of LH-females across the three selection regimes is very likely to represent the difference in the ability of the selection regime males to cause mortality in their mates. We analysed the total number of female deaths per vial across the three regimes. We also analysed rate of mortality. Rate of mortality was derived by regressing cumulative week-wise mortality of ancestral females across all vials against time (in weeks) for each of the nine populations (M1-3, C1-3, F1-3) separately. The least square fit slope was taken as the LH-female mortality rate (mortality per week). This mortality rate could only be calculated for each population. Therefore, the mortality rate of each population was taken as the unit of analysis.

### Measurement of courtship frequency

Courtship frequency of the selection line males were measured after 51–55 generations of selection. On 2nd day after eclosion, virgin selection line males (from one of the 9 populations) were combined with virgin LH-females (5 males: 5 females) in vials with standard food and supplemented with live yeast (2.33 mg per vial). 10 such vials were set up per population. The vials were then returned to the incubator where they were maintained for a day at standard conditions. Courtship frequency was assayed on the two subsequent days during the light phase of the 12 hrs: 12 hrs. L/D cycle. On each day of observation, the vials were placed under uniform over-head lighting at 25°C. Observations started 3 hours after lights-on. Each vial was observed four times a day (total of eight observations over two days). Observations were spaced 1 hour apart. During each observation, a vial was observed for 30 seconds and total number of courtship events (chase, wing-flap, mounting-attempt etc.) was recorded. All vials were numerically coded to ensure a complete blindness of the observer to the identity of the males under observation. We calculated the mean number of courtship per vial per observation from the raw data and this was used as the unit of analysis.

### Measurement of spontaneous locomotor activity

Spontaneous locomotor activity of the selection line males was also assayed after 51–55 generations of selection. Activity of the males was measured using focal sampling method under a set up identical to that followed in courtship frequency assay (described in the previous section). The observation vials (10 vials for each of the nine populations) were divided into four equal sized regions by marking the surface of the vials with a marker. Each region was then numbered for identification. During each observation, a region of a given vial was selected with the help of a random number generator. The individual present in the chosen region was observed. Occasionally more than one individual was present in the selected region and in such situation one out of them was observed. If the chosen region did not have any individual, another random number was generated and the process repeated until a target individual was spotted. Each observation consisted of watching a focal individual for two successive 4-second intervals. If the focal individual showed any displacement within this interval, it was scored as being active. Each vial was observed thrice in a given round of observation. There were four rounds of observations on each observation day. The observations started 3 hours post lights-on in their 12 hrs:12 hrs L/D cycle. Each round of observation was spaced one hour apart. Observer bias was controlled by randomization of the method of selecting the focal individual and by making the assay double blind. An “activity score” was calculated on the basis of the raw data. Mean number of times a given vial was scored as “active” during each observation was calculated. This was averaged across all eight observations for a given vial to derive the “activity score” for that vial. These activity scores (vial values) were taken as the unit of analysis.

### Data analysis

Mean longevity (8–10 replicate per treatment) was analysed using two factor mixed model ANOVA with selection regime as fixed and block as random factor. The two mating status (reproducing and non-reproducing) were analysed separately. Total number of deaths of the ancestral females was analysed using two-factor mixed model ANOVA with selection regime as fixed and block as random factor. The ancestral (LH) female mortality rate was analysed using two-factor mixed model ANOVA with selection regime as fixed factor and Block as random factor. Multiple comparisons were done using paired t-test (paired with respect to blocks) with Dunn-Sidak correction [57].

Dry body weight (section C, 9–10 replicates per population) was analysed using two-factor, mixed model ANOVA with selection regime as fixed factor crossed with random blocks. Data from mate-harm assay were analysed using three factor, mixed model ANOVA with selection regime and exposure status (single mating/continuous exposure) as fixed factors and block as random factor. Courtship frequency and locomotor activity were analysed using two-factor ANOVA with selection regime as fixed factor crossed with random blocks. All multiple comparisons were done using Tukey’s HSD.

As stated before, when assays were spread over multiple generations, all the populations belonging to a given block (e.g., M1, C1 and F1) were assayed together in the same generation. Different blocks were assayed in separate generations. Hence, generation is not modeled as a factor in any of the analyses.

All the analyses were done at α=0.05 level of significant using Statistica (for Windows, version 10, Statsoft). The Cox’s Proportional Hazard analysis if the mortality data was done using R (version 3.0.1), the detail of which is included in the Additional file 1.

## Competing interests

The authors declare that they have no competing interests.

## Authors’ contributions

BN perceived the idea, planned and carried out the experiments, analysed the data and prepared the manuscript. VG, SS, NU and MAS performed the experiments. SZA performed the experiment and analysed the data. NGP perceived the idea, planned the study and prepared the manuscript. All authors read and approved the final version of the manuscript.

## Supplementary Material

Additional file 1 The survivorship analysis of the mortality data: Method and results. Click here for file
