# Supplementary material for: Evolution of mate-harm, longevity and behaviour in male fruit flies subjected to different levels of interlocus conflict
Source: BMC Evol Biol. 2013 Sep 28;13:212. doi: 10.1186/1471-2148-13-212 (PMC3849880; doi:10.1186/1471-2148-13-212)
Supplement: Additional file 1 — The survivorship analysis of the mortality data: Method and results. [file 1471-2148-13-212-S1.docx]

**Additional material**

Survivorship analysis of the mortality data

**Method:**

The reproducing and non-reproducing sets were analysed separately by using Cox’s Proportional hazard model in R (version 3.0.1) [1]. The data was modelled in two different ways – (a) Model-1: considering block as a random factor and selection regime as fixed factor (using R package 'coxme' [2]) and (b) Model-2: only considering selection regime as fixed factor (using R package 'coxphw' [3]). The ‘coxme’ function fits the model:

λ(t) = λ0(t)e^Xβ+Zb^

b ∼ G(0, Σ(θ))

where λ(0) is the baseline hazard function, β is the vector of ﬁxed-eﬀects coeﬃcients and b is the vector of random eﬀects coeﬃcients. G is modelled as Gaussian with mean zero and a variance matrix Σ. ‘Coxphw’ fits the same model without the random effects.

However, ‘coxme’ allows only two levels of the fixed factor. Hence, the three regimes were compared in three pair-wise comparisons (i.e., M vs. C; F vs. C and M vs. F), followed by Dunn-Sidak correction [4]. To test whether there was a significant effect of block on the difference between the two selection regimes under consideration, analyses of deviance between the two models were implemented using the log-likelihood ratios. If block was not found to have a significant effect in this analysis, data from different blocks were pooled and the analysis was performed using selection regime as fixed factor (i.e., Model-2 stated above).

**Results:**

The three regimes were compared pair-wise. Under non-reproducing condition, M-males had significantly higher risk ratio relative to F-males (given by the negative value of the coefficient risk ratio, coxβ, Table A1), consistent across blocks (i.e., non-significant effect of block found in Analysis of deviance, Table A2). The difference between C-regime and other two regimes however, had significant effect of block (Table A2). There was no significant difference between the C and M-regimes (see Model-1, Table A1). For the comparison between C and F-regimes as significant difference was indicated by both Model-1 and Model-2 (Table A1), but there was a significant effect of block (see analysis of deviance, Table A2).

Under reproducing condition, the comparison between M and C-males did not have a significant effect of block (Table A2). Analysis following Model-2 indicated that M-males had significantly higher risk ratio than that of the C-males (i.e., positive coxβ, Table A1). There was significant effect of block on the comparisons between C and F-regimes as well as M and F-regimes (Table A2). There was no significant difference between C and F-regimes (Table A1). The comparison between M and F-regimes was significant in Model-2, and was marginally non-significant in Model-1 (Table A1).

In summary, the survivorship analysis shows that there was a significant amount of across-block heterogeneity. However, non-reproducing M-males consistently showed higher survival risk ratio relative to F-males (and there was no significant effect of block). Reproducing M-males had higher risk ratio relative to C-males (with no significant effect of block). There was a tentative evidence of reproducing M-males having higher risk ratio compared to the F-males of the same mating status.

| **Comparison** | **Model** | **coxβ** | **SE(coxβ)** | **z** | **p** |
| --- | --- | --- | --- | --- | --- |
| **Non-reproducing** | | | | | |
| F vs. C | Model 1 | -0.222 | 0.09 | -2.46 | **0.014** |
|  | Model 2 | -0.198 | 0.051 | -3.88 | **0.0001** |
| M vs. C | Model 1 | 0.208 | 0.215 | 0.97 | 0.33 |
|  | Model 2 | 0.246 | 0.098 | 2.51 | **0.012** |
| F vs. M | Model 1 | -0.639 | 0.103 | -6.23 | **<0.0001** |
|  | Model 2 | -0.639 | 0.103 | -6.23 | **<0.0001** |
| **Reproducing** | | | | | |
| F vs. C | Model 1 | -0.011 | 0.094 | -0.12 | 0.9 |
|  | Model 2 | -0.031 | 0.049 | -0.64 | 0.52 |
| M vs. C | Model 1 | 0.286 | 0.133 | 2.14 | 0.032 |
|  | Model 2 | 0.264 | 0.0977 | 2.7 | **0.0069** |
| F vs. M | Model 1 | -0.308 | 0.133 | -2.32 | 0.02 |
|  | Model 2 | -0.322 | 0.101 | -3.18 | **0.0015** |

**Table S1:** Results of the pair-wise analysis of the survivorship data using Cox's Proportional Hazard model. Model-1: Selection regime as fixed factor, block as random factor; Model-2: Selection regime as fixed factor. coxβ is the coefficient of the risk ratio between the two regimes under comparison (in the order mentioned in "comparisons" column). A negative value of the coxβ means that the later regime in the comparison has greater risk ratio, i.e., lower survivorship, and vice versa.

| **Comparison** | **Model** | **log-likelihood ratio** | **Chi-sq** | **DF** | **p** |
| --- | --- | --- | --- | --- | --- |
| F vs. C | Model 1 | -1969.4 |  |  |  |
|  | Model 2 | -1975 | 11.306 | 3 | **0.01** |
| M vs. C | Model 1 | -2124.6 |  |  |  |
|  | Model 2 | -2129.8 | 10.458 | 3 | **0.015** |
| F vs. M | Model 1 | 2023.1 |  |  |  |
|  | Model 2 | 2023.1 | 0.0013 | 3 | 1 |
| F vs. C | Model 1 | -2115.5 |  |  |  |
|  | Model 2 | -2120.6 | 10.205 | 3 | **0.001** |
| M vs. C | Model 1 | -2158.6 |  |  |  |
|  | Model 2 | -2159.6 | 1.954 | 3 | 0.16 |
| F vs. M | Model 1 | -2042.4 |  |  |  |
|  | Model 2 | -2049.5 | 14.085 | 3 | **0.0002** |

**Table S2:** Results of analysis of deviance. This analysis compared the results of Cox’s Proportional Hazard Model-1 and Model-2. Model-1: Selection regime as fixed factor, block as random factor; Model-2: Selection regime as fixed factor.

**Literature Cited:**

1. R Core Team: **R: A language and environment for statistical computing. R Foundation for Statistical Computing, Vienna, Austria.** (2013), [URL http://www.R-project.org/]
2. Therneau T: **Coxme: Mixed Effects Cox Models.** R package version 2.2-3 (2012), [http://CRAN.R-project.org/package=coxme]
3. R by Meinhard Ploner and Fortran by Georg Heinze (2012). **coxphw: Weighted Cox regression**. R package version 2.12. [http://CRAN.R-project.org/package=coxphw]
4. Sokal RR, Rohlf FJ: Biometry, 3^rd^ ed. NY, W.H. Freeman and Company, 1995:238-240.
